# Off pump reduction aortoplasty in ascending aortic aneurysm

**DOI:** 10.1186/1749-8090-8-S1-O14

**Published:** 2013-09-11

**Authors:** G Coronella, P Pepino, E Pezzella, A Contaldo, R Provenzano, P Oliviero, S Di Maio, V Schiavone, S Giordano, M Monaco

**Affiliations:** 1 Department of Cardiothoracic Surgery, PO Pineta Grande, Castel Volturno, Italy; 2 Department of Cardiology, PO Pineta Grande, Castel Volturno, Italy; 3 Department of Anesthesiology and Intensive Care, PO Pineta Grande, Castel Volturno, Italy

## Background

Sixty percent of thoracic aortic aneurism involve the aortic root/or ascending aorta. The optimal timing of surgical repair remains uncertain despite most surgeons prefer to treat the aneurysm at a diameter of ≥5,5cm. This paper describes our off pump option for the treatment of isolated asending aorta aneurism with a diameter between 5,0 and 5,5 cm.

## Methods

Three patients with a story of ascending aorta aneurysm were selected from October 2012 to December 2012. Trans thoracic echocardiography, angio CT scan and a coronarography were performed during the pre op evaluation. The surgical procedure was performed on beating heart in off pump using a low dose of heparin (5000 UI). A side clamp was used to plicate longitudinally the ascending aorta at the level of maximum dilatation from the sino tubular junction up to the troncus anonymous with controlled hypotension (70 mmHg).The plication was performed with a double layer of 3/0 prolene suture reinforced with two strips of Teflon along the plication. Furthermore, an opened longitudinally 32 mm Dacron tube was wrapped around the ascending aorta and sutured to the plication with a double 3/0 prolene suture.

## Results

All patients were estubated in an hour after the operation. No major complications were observed. A post op echocardiography and an angio CT scan were performed at the discharge showing a reduction of the ascending aorta aneurysm with a maximum diameter of 3,5 cm.

## Conclusion

The procedure previously described is an easy procedure without cardiopulmonary bypass that according to the Laplace low (the wall tension is the result of the pressure for radius) reducing the radius of the aorta, allows to reduce the wall tension. Furthermore the bending of the ascending aorta reduces the possibility of rupture of the aorta. This is a preliminary experience that despite initially results are encouraging (CT scan after six month doesn't show any changes) must be controlled with a mid term follow up.

